# Reproductive outcomes of dual trigger therapy with GnRH agonist and hCG versus hCG trigger in women with diminished ovarian reserve: a retrospective study

**DOI:** 10.1186/s12958-024-01211-z

**Published:** 2024-04-02

**Authors:** Kai Chen, Chunmei Zhang, Lixue Chen, Yue Zhao, Hongzhen Li

**Affiliations:** 1 State Key Laboratory of Female Fertility Promotion, Center for Reproductive Medicine, Department of Obstetrics and Gynecology, Peking University Third Hospital, HaiDian District, No. 49 HuaYuan North Road, Beijing, 100191 China; 2 National Clinical Research Center for Obstetrics and Gynecology, Peking University Third Hospital, Beijing, 100191 China; 3 Key Laboratory of Assisted Reproduction, Ministry of Education, Beijing, 100191 China; 4 Beijing Key Laboratory of Reproductive Endocrinology and Assisted Reproductive Technology, Beijing, 100191 China; 5 Research Units of Comprehensive Diagnosis and Treatment of Oocyte Maturation Arrest, Chinese Academy of Medical Sciences, Beijing, 100191 China; 6 National Clinical Key Specialty Construction Program, P. R. China (2023), Beijing, China

**Keywords:** Diminished ovarian reserve, Dual trigger, hCG trigger, Mild stimulation, Pregnancy outcomes

## Abstract

**Background:**

Diminished ovarian reserve (DOR) is one of the obstacles affecting the reproductive outcomes of patients receiving assisted reproductive therapy. The purpose of this study was to investigate whether dual trigger, including gonadotropin‐releasing hormone agonist (GnRHa) and human chorionic gonadotropin (hCG), can improve pregnancy outcomes in patients with DOR undergoing in vitro fertilization (IVF) cycles using mild stimulation protocols.

**Methods:**

A total of 734 patients with DOR were included in this retrospective study. Patients were divided into a recombinant hCG trigger group and a dual trigger group (hCG combined with GnRHa) according to the different trigger drugs used. The main outcome measures included the number of oocytes retrieved, the fertilization rate, the number of transferable embryos, the implantation rate, the clinical pregnancy rate, the miscarriage rate, the live birth rate (LBR), and the cumulative live birth rate (CLBR). Generalized linear model and logistic regression analyses were performed for confounding factors.

**Results:**

There were 337 cycles with a single hCG trigger and 397 cycles with dual trigger. The dual trigger group demonstrated significantly higher numbers of retrieved oocytes [3.60 vs. 2.39, adjusted β = 0.538 (0.221–0.855)], fertilized oocytes [2.55 vs. 1.94, adjusted β = 0.277 (0.031–0.523)] and transferable embryos [1.22 vs. 0.95, adjusted β = 0.162 (-0.005–0.329)] than did the hCG trigger group, whereas no significant difference in the fertilization rate was observed between the two groups. Moreover, the embryo transfer cancellation rate (35.5% vs. 43.9%) was obviously lower in the dual trigger group. Among the fresh embryo transfer cycles, the implantation rate, clinical pregnancy rate, miscarriage rate and live birth rate were similar between the two groups. After controlling for potential confounding variables, the trigger method was identified as an independent factor affecting the number of oocytes retrieved but had no significant impact on the CLBR.

**Conclusions:**

Dual triggering of final oocyte maturation with hCG combined with GnRHa can significantly increase the number of oocytes retrieved in patients with DOR but has no improvement effect on the implantation rate, clinical pregnancy rate or LBR of fresh cycles or on the CLBR.

**Supplementary Information:**

The online version contains supplementary material available at 10.1186/s12958-024-01211-z.

## Background

Diminished ovarian reserve (DOR) is a common reproductive endocrine disease in women of reproductive age, and its incidence in infertile women is approximately 10% [1]. DOR is characterized by an increase in the serum level of follicle-stimulating hormone (FSH) and a decrease in the serum level of anti-Müllerian hormone (AMH) and the antral follicle count (AFC). In addition, DOR is often accompanied by a reduction in the quality and quantity of oocytes, leading to unsatisfactory outcomes in assisted reproductive therapy (ART), such as fewer blastocyst transfer cycles and a higher miscarriage rate [2, 3], which has caused great psychological and economic pressure. Therefore, how to optimize the outcomes of ART in patients with DOR has become a widespread concern of researchers.

Previous studies have shown that both letrozole and clomiphene could increase the release of gonadotropin-releasing hormone (GnRH) by affecting the feedback regulation of GnRH release by estrogen, thereby reducing the use of gonadotropins (Gn) in ART patients [4, 5]. Therefore, mild stimulation protocols [6], including letrozole or clomiphene, are increasingly used in the process of ART because of their good efficacy and relatively low cost. Another critical step at the end of controlled ovarian hyperstimulation (COH) is triggering final oocyte maturation. Human chorionic gonadotropin (hCG) is a traditional drug used to trigger ovulation [7]. It has similar biological activity to luteinizing hormone (LH) and can simulate the natural LH surge, thereby promoting the maturation of oocytes by restoring oocyte meiosis [8, 9]. In addition, several researchers have reported that the GnRH agonist (GnRHa) is a surrogate for the ovulation trigger. GnRHa can induce surges in FSH and LH simultaneously, similar to the changes observed under physiological conditions [10]. Moreover, elevated FSH levels can further promote the maturation of oocytes by inducing the formation of LH receptors (LHRs) in luteinized granulosa cells [11]. Previous studies have indicated that dual trigger (hCG and GnRHa) could not only improve the clinical pregnancy rate and LBR but also enhance the quality of embryos from patients with a normal ovarian response [10, 12, 13].

Previous studies have suggested that mild stimulation protocols could significantly increase the proportion of high-quality oocytes in patients with DOR [5]. Researchers are still uncertain whether dual trigger can improve the reproductive outcomes of patients with DOR, especially patients undergoing in vitro fertilization (IVF) treatments via mild stimulation protocols. Lin MH et al. and Chern CU et al. suggested that dual trigger could increase the number of retrieved oocytes and the LBR in patients with DOR [14, 15]. In contrast, Hong YH et al. and Ren YM et al. suggested that dual trigger had no significant effect on improving the reproductive outcomes of patients with DOR [16, 17]. Therefore, our study aimed to conduct a retrospective analysis with a larger sample size to investigate whether dual trigger could improve the reproductive outcomes of patients with DOR undergoing IVF cycles using mild stimulation protocols.

## Methods

### Study design and participants

This study included 734 patients with DOR who underwent IVF/ICSI at the Center of Peking University Third Hospital from March 2019 to September 2020. DOR was diagnosed in accordance with the Bologna criteria [18].

### Ovarian stimulation protocols

Mild stimulation with clomiphene/letrozole combined with Gn was used for ovarian stimulation. The detailed protocol was as follows: clomiphene 50–100 mg or letrozole 2.5–5 mg or combined was given orally from day 2 to 6 of the menstrual cycle, while Gn was injected on the third day of menstruation with an initial dose between 150 IU-225 IU until the trigger day, adjusted depending on the ovarian response. Gonadotropin-releasing hormone antagonist (GnRH-ant) was added according to the follicle diameter and estradiol level to prevent a premature LH peak. Recombinant human choriogonadotropin alpha solution for injection (rHCG, Ovidrel, Merck Serono, Germany) (250 µg) or a combination of rHCG and GnRHa (triptorelin acetate for injection; Ipsen Pharma (Biotech), France) (0.2 mg) was used at the same time when at least the diameter of 2 dominant follicles reached 18 mm. Oocyte retrieval was performed via transvaginal ultrasound 36 h later. Conventional IVF or intracytoplasmic sperm injection (ICSI) was conducted, and two embryos or one blastocyst were transferred on the third or fifth day after oocyte retrieval. Serum β-HCG was measured 14 days after embryo or blastocyst transfer. Luteal support was maintained until 10 weeks of gestation if conception occurred.

### Outcome measures

The primary outcome parameter was the CLBR. CLBR has been suggested as a suitable way of reporting the success of an IVF program, which means the total chance rate of live birth of each retrieval cycle after all the embryos obtained are transferred. The secondary outcome parameters included the number of retrieved oocytes, fertilization rate, number of transferable embryos, embryo transfer cancellation rate, implantation rate, miscarriage rate, clinical pregnancy rate and LBR in the fresh embryo transfer cycles. The embryo transfer cancellation rate was defined as the number of cycles in which no oocytes were retrieved or no embryos were available divided by the total number of cycles. Clinical pregnancy was defined as a positive pregnancy blood test followed by the presence of a gestational sac on transvaginal ultrasound 30 days after embryo or blastocyst transfer. Miscarriage and live birth were defined as described previously [19].

### Statistical analysis

SPSS (version 26.0) was used for the data analysis. The normality of the data was checked using the Kolmogorov–Smirnov test, box plot graphs, histograms and quantile–quantile plot graphs. The differences in continuous variables between two groups were analyzed by an independent Student’s *t* test and the Mann‒Whitney *U* test. Categorical variables were analyzed by Pearson’s chi-square test, the continuity correction chi-square test and Fisher's exact test. Subsequently, generalized linear models adjusted for potential confounders were applied to evaluate the associations between trigger methods and embryological outcomes. The odds ratio (OR) and 95% confidence interval (CI) obtained from binary logistic regression models were used to compare the cancellation rate, clinical pregnancy rate, and CLBR between the two groups. The variables in the regression models included the method of triggering, body mass index (BMI), the serum levels of FSH and AMH, the dosage of Gn, mild stimulation and fertilization method. In addition, to further adjust the baseline characteristics of the two groups of patients, we conducted case control matching between the two groups of patients based on BMI, the serum levels of FSH and AMH, and the dosage of Gn. All the data are expressed as the mean ± standard deviation (SD), median (interquartile range [IQR]) or number (n) and frequency (%), and *P* < 0.05 was considered to indicate a significant difference.

## Results

### Patient characteristics

**Fig. 1:**
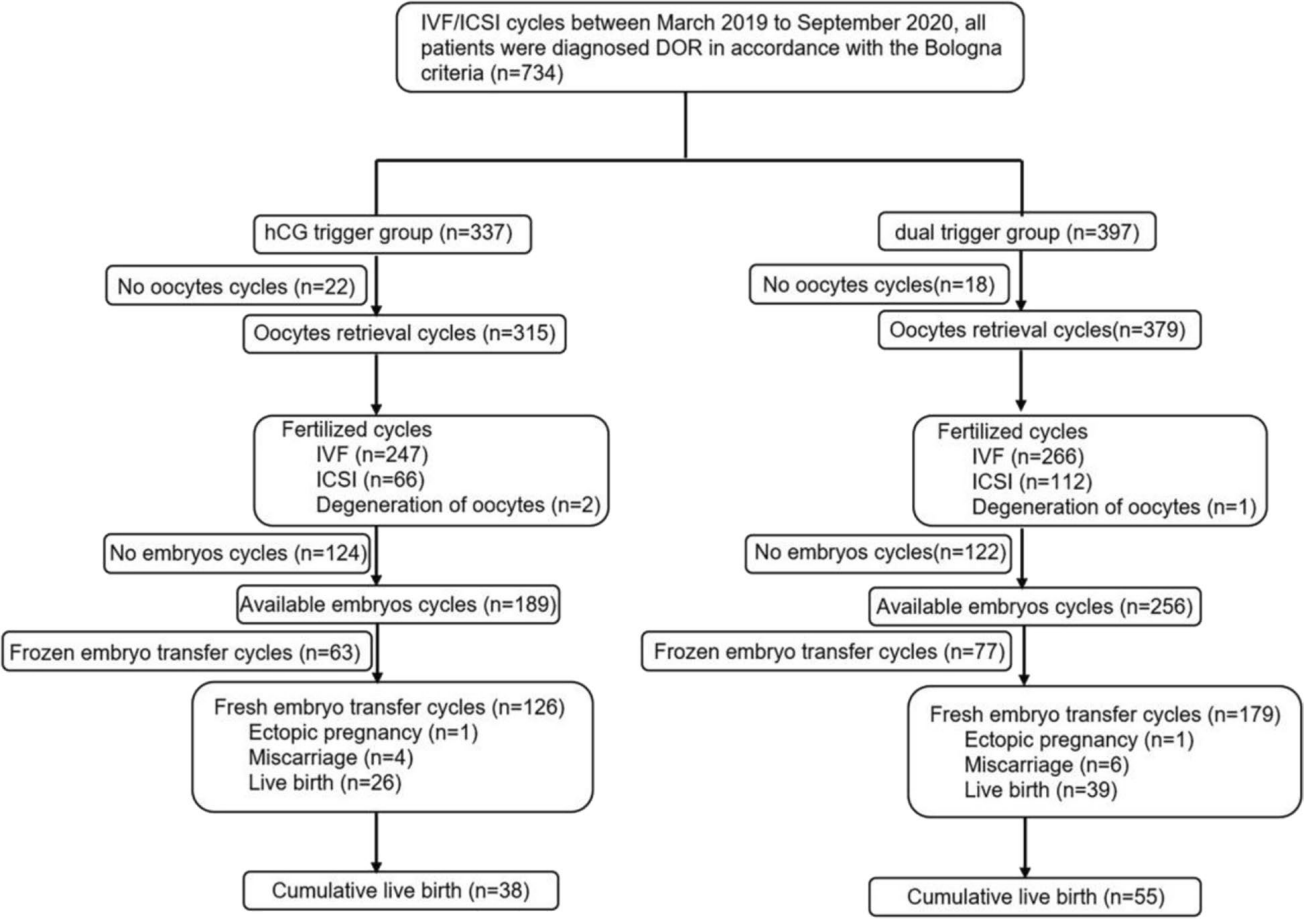
The study flowchart. The detailed inclusion/exclusion process and the number of cycles at each stage

A total of 734 patients with DOR received different ovulation triggers, including 337 cycles in the hCG trigger group and 397 cycles in the dual trigger group. Figure 1 shows the flow of the entire study. The baseline characteristics of the patients are indicated in Table 1. There was no significant difference in the age of the patients between the two groups. The body mass index (BMI) and the serum levels of FSH and LH in the patients in the dual trigger group were lower than those in the hCG trigger group, whereas the serum level of AMH was higher in the dual trigger group. Moreover, we also found that the dosage of Gn and the serum level of estradiol on the trigger day were significantly increased, while the serum level of LH on the trigger day was decreased in the dual trigger group. In addition, we also observed that the proportions of different drugs used for mild stimulation were significantly different between the two groups, but when we performed case control matching, the difference in the proportion of drugs disappeared. Moreover, BMI, the serum levels of FSH and AMH and the dosage of Gn were not significantly different between the two groups (Supplementary Table 1).

**Table 1:** Characteristics of the patients in the two groups

	hCG trigger	Dual trigger	*p* value	N
	***N*** ** = 337**	***N*** ** = 397**		
The baseline characteristics				
Age (year)	37. 4 ± 5.11	37.4 ± 5.07	0.963^a^	734
BMI (kg/m^2^)	23.1 ± 3.37	22.5 ± 3.31	0.014^a^	733
FSH (IU/L)	11.8 ± 6.08	10.2 ± 5.25	< 0.001^a^	711
E2 (pmol/L)	189 ± 119	179 ± 98.7	0.192^a^	712
P (nmol/L)	1.04 (0.69, 1.49)	1.05 (0.75, 1.42)	0.989^b^	682
PRL (ng/ml)	12.6 ± 7.78	12.6 ± 6.94	0.922^a^	623
LH (IU/L)	3.80 (2.58, 5.58)	3.40 (2.35, 5.05)	0.016^b^	712
T (nmol/l)	0.69 (0.69, 0.69)	0.69 (0.69,0.69)	0.915^b^	587
A (nmol/L)	4.39 (3.03, 6.38)	4.66 (3.50, 6.64)	0.160^b^	515
AMH (ng/mL)	0.41 (0.18, 0.69)	0.52 (0.28, 0.96)	< 0.001^b^	717
Dosage of Gn (IU)	1739 ± 912	2075 ± 1001	< 0.001^a^	734
Duration of stimulation (d)	11.9 ± 2.66	11.5 ± 2.13	0.058^a^	725
Hormone levels on trigger day				
LH (IU/L)	4.05 (2.11, 7.44)	3.07 (1.87, 6.06)	< 0.001^b^	727
E2 (pmol/L)	1586 (946.00, 2516.75)	1744 (1077.25, 3099.25)	0.005^b^	728
P (nmol/L)	1.47 (1.05, 2.09)	1.54 (1.01, 2.39)	0.202^b^	727
Previous stimulation cycles and cancellations	1.4 ± 1.18	1.20 ± 1.28	0.071^a^	734
Mild stimulation			0.006^c^	734
LE	195 (57.9%)	274 (69.0%)		
CC	67 (19.9%)	54 (13.6%)		
LE + CC	75 (22.3%)	69 (17.4%)		

*BMI* Body mass index, *FSH* Follicle-stimulating hormone, *PRL* Prolactin, *LH *Luteinizing hormone, *AMH* Anti-Müllerian hormone, *LE* Letrozole, *CC* Clomiphene

Data are presented as the mean ± SD or median (IQR)

^a^Independent Student’s t test

^b^Mann‒Whitney U test

^c^Pearson’s chi-squared test

### Outcomes of ovarian stimulation

After ovarian stimulation, there were 40 cycles without oocytes retrieved. In terms of the number of IVF/ICSI cycles, compared with those in the hCG trigger group, the numbers of oocytes retrieved (3.60 vs. 2.39, *P* < 0.001), fertilized oocytes (2.55 vs. 1.94, *P* < 0.001), 2-pronuclei (2PN) embryos (2.02 vs. 1.52, *P* < 0.001) and transferable embryos (1.22 vs. 0.95, *P* = 0.001) in dual trigger group were significantly higher. The fertilization rate and cleavage rate of the 2PN embryos and the embryo developmental stage at transfer were similar between the two groups. Although there was a difference in the method of fertilization between the two groups (Table 2), the difference in the method of fertilization disappeared when we performed case control matching (Supplementary Table 1).

**Table 2:** Comparison of IVF-ICSI outcomes between the two groups

	hCG trigger	Dual trigger	*p* value	*N*
	***N* = 337**	***N* = 397**		
Number of cycles without oocytes retrieved	22/337 (6.53%)	18/397 (4.53%)	0.236^b^	734
No. of retrieved oocytes	2.39 ± 1.90	3.60 ± 2.73	< 0.001^a^	734
Fertilization method			0.011^b^	691
IVF	247 (78.9%)	266 (70.4%)		
ICSI	66 (21.1%)	112 (29.6%)		
No. of fertilized oocytes	1.94 ± 1.46	2.55 ± 1.82	< 0.001^a^	694
No. of 2PN embryos	1.52 ± 1.34	2.02 ± 1.60	< 0.001^a^	694
Fertilization rate (%)				
IVF	70.8 ± 37.7	73.9 ± 34.8	0.340^a^	513
ICSI	73.2 ± 42.2	78.4 ± 34.8	0.401^a^	178
Cleavage rate of 2PN embryos	70.0 ± 39.7	74.8 ± 35.5	0.103^a^	683
No. of transferable embryos	0.95 ± 0.98	1.22 ± 1.13	0.001^a^	694
Embryo developmental stage at transfer			0.902^c^	305
D3	123 (96.9%)	174 (97.8%)		
D5	4 (3.1%)	4 (2.2%)		

*IVF* In vitro fertilization, *ICSI *Intracytoplasmic sperm injection, *2PN* 2-pronuclei, *D3* cleavage embryos, *D5* blastocyst

Data are presented as the mean ± SD or as n (%)

^a^Independent Student’s t test

^b^Pearson’s chi-squared test

^c^Continuity Correction chi-square test

### Pregnancy outcomes

The pregnancy outcomes of the two groups are shown in Table 3. The embryo transfer cancellation rate was significantly lower in the dual trigger group than in the hCG trigger group (35.5% vs. 43.9%, *P* = 0.020). Moreover, during the fresh embryo transfer cycles, there were no significant differences in the implantation rate (16.6% vs. 17.6%, *P* = 0.784), clinical pregnancy rate (25.7% vs. 24.6%, *P* = 0.828), miscarriage rate (13.3% vs. 13.3%, *P* = 1.000) or LBR (21.9% vs. 20.8%, *P* = 0.817) between the two groups. In addition, the CLBR (21.6% vs. 20.2%, *P* = 0.729) was also similar between the two groups.

**Table 3:** Comparison of pregnancy outcomes between the two groups

	hCG trigger	Dual trigger	*p* value	*N*
	***N* = 337**	***N* = 397**		
Embryo transfer cancellation rate (%)	148/337 (43.9%)	141/397 (35.5%)	0.020^b^	734
Number of embryo transfer cycles			0.465^b^	445
Fresh embryo transfer cycles	126 (66.7%)	179 (69.9%)		
Frozen embryo transfer cycles	63 (33.3%)	77 (30.1%)		
Fresh embryo transfer cycles				
Implantation rate (%)	17.6 ± 33.8	16.6 ± 30.9	0.784^a^	303
Clinical pregnancy rate (%)	31/126 (24.6%)	46/179 (25.70%)	0.828^b^	305
Ectopic pregnancy rate (%)	1/31 (3.23%)	1/46 (2.17%)	1.000^d^	77
Miscarriage rate (%)	4/30 (13.3%)	6/45 (13.3%)	1.000^c^	75
LBR (%)	26/125 (20.8%)	39/178 (21.9%)	0.817^b^	303
CLBR (%)	38/188 (20.2%)	55/255 (21.6%)	0.729^b^	443

LBR live birth rate. CLBR cumulative live birth rate

Data are presented as the mean ± SD or as n (%)

^a^Independent Student’s t test

^b^Pearson’s chi-squared test

^c^Continuity Correction chi-square test

^d^Fisher’s exact test

### Multivariate regression analysis for IVF-ICSI outcomes

Since the patients in the dual trigger group had lower serum FSH levels and higher AMH levels, suggesting that they might have better ovarian function than patients in the hCG trigger group did. Furthermore, the drugs used for mild stimulation and the fertilization method used also differed between the two groups, and these differences in baseline characteristics may have affected IVF/ICSI outcomes between the two groups. In addition, a higher dosage of Gn can influence the outcomes of ovarian stimulation. Therefore, we further performed regression analyses to exclude the interference of these confounding factors. After adjusting for BMI, the serum levels of FSH and AMH, the dosage of Gn, mild stimulation and fertilization method, compared with patients in the hCG trigger group, patients in the dual trigger group still had a significantly increased number of retrieved oocytes [adjusted β = 0.538 (0.221–0.855), *P* = 0.001] and fertilized oocytes [adjusted β = 0.277 (0.031–0.523), *P* = 0.027]; however, there were no significant differences in the number of transferable embryos [adjusted β = 0.162 (-0.005–0.329), *P* = 0.057] or cancellation rate [adjusted OR = 1.168 (0.828–1.648), *P* = 0.377] between the two groups after adjusting for confounding factors (Tables 4 and 5). In addition, the trigger method was not significantly associated with the clinical pregnancy rate in fresh embryo transfer cycles or with the CLBR, regardless of whether these confounding factors were adjusted for (Table 5). When we performed case control matching, we were still able to observe a higher number of retrieved oocytes in the dual trigger group. However, the difference in the number of fertilized oocytes between the two groups disappeared (Supplementary Table 2), suggesting that the association between the number of fertilized oocytes and the trigger method needs to be further verified.

**Table 4:** Multivariate analysis of the association between trigger methods and embryological outcomes

Outcomes	β (95% CI)	*p* value	Adjusted β^a^ (95% CI)	*p* value
**No. of oocytes retrieved**				
hCG trigger	Ref.		Ref.	
Dual trigger	1.210 (0.863–1.556)	0.000	0.538 (0.221–0.855)	0.001
**No. of transferable embryos**				
hCG trigger	Ref.		Ref.	
Dual trigger	0.267 (0.108–0.426)	0.001	0.162 (-0.005–0.329)	0.057
**No. of fertilized oocytes**				
hCG trigger	Ref.		Ref.	
Dual trigger	0.609 (0.360–0.859)	0.000	0.277 (0.031–0.523)	0.027

*CI* Confidence interval

^a^Adjusted for BMI, FSH, AMH, dosage of Gn, mild stimulation and fertilization method

**Table 5:** Multivariate analysis of the association between trigger methods and pregnancy outcomes

Outcomes	OR (95% CI)	*p* value	Adjusted OR^a^ (95% CI)	*p* value
**Cancellation rate**				
hCG trigger	Ref.		Ref.	
Dual trigger	1.422 (1.056–1.914)	0.020	1.168 (0.828–1.648)	0.377
**Clinical pregnancy rate**				
hCG trigger	Ref.		Ref.	
Dual trigger	1.060 (0.626–1.794)	0.828	0.997 (0.563–1.764)	0.991
**Cumulative live birth rate**				
hCG trigger	Ref.		Ref.	
Dual trigger	1.086 (0.682–1.727)	0.729	1.030 (0.620–1.711)	0.909

*OR* odds ratio, *CI* confidence interval

^a^Adjusted for BMI, FSH, AMH, dosage of Gn, mild stimulation and fertilization method

## Discussion

In recent years, the number of women with DOR has been constantly increasing with delayed childbearing. Improving the pregnancy outcomes of patients with DOR has become the focus of increased amounts of attention. Previous studies have shown that mild stimulation protocols have the advantages of low cost and limited stimulation [5], so they are increasingly used in clinical practice. However, DOR is often accompanied by an age-related decrease in oocyte quality; therefore, promoting the maturation of oocytes to obtain more available embryos is critical. Triggering final oocyte maturation with a combination of hCG and GnRHa has recently been suggested to improve IVF/ICSI treatment outcomes. GnRHa can induce a preovulatory LH/FSH surge at the same time. A surge in FSH levels during IVF-ICSI cycles can not only restore the meiosis of some oocytes [20] but also cause LH receptor formation, nuclear maturation and cumulus expansion in luteinized granulosa cells [21]. A randomized controlled study and a retrospective cohort study demonstrated that dual trigger significantly improved the pregnancy rate and LBR in patients with a normal ovarian response [12, 22]. In addition, Hong YH et al. reported that dual trigger could significantly increase the number of mature oocytes in patients with breast and endometrial cancers treated with letrozole [16].

Our findings provide reliable evidence on whether patients with DOR should use dual trigger in mild stimulation IVF/ICSI protocols. In the present study, dual trigger significantly increased the number of retrieved oocytes but did not significantly improve the number of transferable embryos, implantation rate, clinical pregnancy rate or LBR in fresh embryo transfer cycles, as well as the CLBR. Previous studies have shown that the expression of amphiregulin and epiregulin in cumulus granulosa cells is significantly increased in patients with dual trigger [23]. Amphiregulin and epiregulin are ligands of epidermal growth factor receptor (EGFR), and elevated EGFR protein expression can induce EGFR activation by increasing LH levels, thereby promoting oocyte maturation and cumulus cell expansion in the ovary [24, 25] and leading to an increased number of retrieved oocytes.

On the other hand, dual trigger not only increases the number of injections for patients, but also aggravates the economic burden with increased expenses by 30 dollars per person. Although this increased expenditure is relatively small compared to the total cost of IVF, considering the limited role of dual trigger in improving the pregnancy outcomes of patients with DOR as our study indicated, patients with DOR do not seem to benefit from dual trigger. Ren YM et al. compared the pregnancy outcomes of 181 patients with DOR and reported no significant changes in the implantation rate, miscarriage rate or CBLR between the dual trigger group and the hCG trigger group, which is consistent with our findings [17]. However, Lin MH et al. proposed the opposite opinion: dual trigger could significantly improve the clinical pregnancy rate and LBR of patients with DOR, possibly because GnRHa given before ovulation could maintain biological activity during the embryo implantation period due to its longer half-life [14]. One study confirmed that injection of GnRHa during the luteal phase can significantly improve the implantation rate and clinical pregnancy rate of patients [26], and these positive effects seem to be closely related to the regulatory effects of GnRHa on endometrial GnRH receptors, endometrial receptivity, endometrial decidualization and trophoblast invasion [26–29]. However, it is still uncertain whether GnRHa injected at the time of triggering ovulation can still have high biological activity during the luteal phase, and further research is needed to confirm this phenomenon.

In general, although patients in the dual trigger group had better ovarian function, which may have a certain impact on our research conclusions, we used a regression model to correct for the confounding effects of BMI, the serum levels of FSH and AMH and dosage of Gn, which were significantly different between the two groups. On the other hand, case control matching was performed between the two groups for the above four indicators to further eliminate the effects of inconsistent ovarian function between the two groups. This makes our research conclusions more reliable, and this is one of the advantages of our research.

This study has several limitations. First, this study evaluated the outcomes of only patients who underwent hCG trigger or dual trigger, while patients who received a single GnRHa trigger were not included. Second, our study was a retrospective cohort study, and selection bias may have occurred. Thus, a double-blind randomized controlled study is needed to further confirm the efficacy of dual trigger in patients with DOR under mild stimulation protocols.

In conclusion, compared with the hCG trigger, dual triggering of final oocyte maturation with hCG combined with GnRHa can significantly increase the number of oocytes retrieved in patients with DOR but has no obvious improvement on pregnancy outcomes, especially the CLBR.

### Supplementary Information

**Supplementary Material 1.**

## Data Availability

The data used and analyzed during the present study are available from the database of the Center for Reproductive Medicine in Peking University Third Hospital upon reasonable request.

## Abbreviations

DOR: Diminished ovarian reserve
Hcg: Human chorionic gonadotropin
GnRH: Gonadotropin-releasing hormone
FSH: Follicle-stimulating hormone
AMH: Anti-Müllerian hormone
PRL: Prolactin
LH: Luteinizing hormone
Gn: Gonadotropins
COH: Controlled ovarian hyperstimulation
IVF: In vitro fertilization
ICSI: Intracytoplasmic sperm injection
2PN: 2-Pronuclei
LBR: Live birth rate
CLBR: Cumulative live birth rate
BMI: Body mass index
