# Supplementary material for: Reproductive outcomes of dual trigger therapy with GnRH agonist and hCG versus hCG trigger in women with diminished ovarian reserve: a retrospective study
Source: Reprod Biol Endocrinol. 2024 Apr 2;22:35. doi: 10.1186/s12958-024-01211-z (PMC10985881; doi:10.1186/s12958-024-01211-z)
Supplement: Supplementary file 1 — Supplementary Material 1. [file 12958_2024_1211_MOESM1_ESM.docx]

**Supplementary table 1: Characteristics of the patients in the two groups.**

|  | hCG trigger | Dual trigger | *p* value | N |
| --- | --- | --- | --- | --- |
|  | N=126 | N=126 |  |  |
| The baseline characteristics | | | | |
| Age (year) | 37.2 ± 5.09 | 38.2 ± 5.11 | 0.130^a^ | 252 |
| BMI (kg/m^2^) | 22.7 ± 2.72 | 22.7 ± 2.72 | 0.952^a^ | 252 |
| FSH (IU/L) | 9.7 ± 3.63 | 9.7 ± 3.63 | 0.939^a^ | 252 |
| E2 (pmol/L) | 209.7 ± 120.38 | 168.2 ± 88.93 | 0.002^a^ | 252 |
| P (nmol/L) | 1.12 ± 0.53 | 1.16 ± 0.60 | 0.648^a^ | 241 |
| PRL (ng/ml) | 11.8 ± 6.76 | 12.3 ± 6.18 | 0.548^a^ | 223 |
| LH (IU/L) | 3.40 (2.32, 4.62) | 3.28 (2.20, 4.86) | 0.857^b^ | 252 |
| T (nmol/l) | 0.69 (0.69, 0.75) | 0.69 (0.69, 0.69) | 0.361^b^ | 205 |
| A (nmol/L) | 4.8 ± 3.61 | 5.1 ± 2.60 | 0.541^a^ | 177 |
| AMH (ng/mL) | 0.5 ± 0.36 | 0.5 ± 0.37 | 0.499^a^ | 717 |
| Dosage of Gn (IU) | 1779.4 ± 696.39 | 1772.8 ± 693.43 | 0.940^a^ | 252 |
| Duration of stimulation (d) | 11.8 ± 2.20 | 11.4 ± 2.18 | 0.203^a^ | 248 |
| Hormone levels on trigger day | | | | |
| LH (IU/L) | 3.95 (2.04, 7.44) | 3.05 (1.80, 6.16) | 0.025^b^ | 248 |
| E2 (pmol/L) | 2374.9 ± 1755.63 | 2219.2 ± 1859.45 | 0.498^a^ | 248 |
| P (nmol/L) | 1.51 (1.12, 2.23) | 1.45 (0.88, 2.44) | 0.286^b^ | 247 |
| Previous stimulation cycles and cancellations | 1.4 ± 1.11 | 1.2 ± 1.22 | 0.053^a^ | 252 |
| Mild stimulation |  |  | 0.187^c^ | 252 |
| LE | 72 (57.1%) | 80 (63.5%) |  |  |
| CC | 34 (27.0%) | 22 (17.5%) |  |  |
| LE+CC | 20 (15.9%) | 24 (19.0%) |  |  |
| Fertilization method |  |  | 0.918^c^ | 236 |
| IVF | 95 (81.2%) | 96 (80.7%) |  |  |
| ICSI | 22 (18.8%) | 23 (19.3%) |  |  |
| Embryo developmental stage at transfer |  |  | 0.679^d^ | 105 |
| D3 | 52 (94.5%) | 49 (98.0%) |  |  |
| D5 | 3 (5.5%) | 1 (2.0%) |  |  |

Note: Data are presented as the mean ± SD or median (IQR) or n (%). ^a^ Independent Student’s t test. ^b^ Mann‒Whitney U test. ^c^ Pearson’s chi-squared test. ^d^ Continuity Correction chi-square test. BMI: body mass index, FSH: follicle-stimulating hormone, PRL: prolactin, LH: luteinizing hormone, AMH: anti-Müllerian hormone, LE: letrozole, CC: clomiphene, D3: cleavage embryos, D5: blastocyst.

**Supplementary table 2: Comparison of IVF-ICSI outcomes between the two groups.**

|  | hCG trigger | Dual trigger | *p* value | N |
| --- | --- | --- | --- | --- |
|  | N=126 | N=126 |  |  |
| No. of retrieved oocytes | 2.57 ± 1.78 | 3.08 ± 2.22 | 0.046^a^ | 252 |
| No. of fertilized oocytes | 2.18 ± 1.50 | 2.40 ± 1.73 | 0.290^a^ | 236 |
| No. of transferable embryos | 1.08 ± 0.96 | 1.15 ± 1.15 | 0.591^a^ | 236 |
| Embryo transfer cancellation rate (%) | 46/126 (36.5%) | 53/126 (42.1%) | 0.367^b^ | 252 |
| Fresh embryo transfer cycles |  |  |  |  |
| Clinical pregnancy rate (%) | 12/55 (21.8%) | 17/52 (32.7%) | 0.206^b^ | 107 |
| CLBR (%) | 18/79 (22.8%) | 15/72 (20.8%) | 0.772^b^ | 151 |

Note: Data are presented as the mean ± SD or as n (%). ^a^ Independent Student’s t test. ^b^ Pearson’s chi-squared test. CLBR: cumulative live birth rate.
